# Functional assays to assess the therapeutic potential of extracellular vesicles

**DOI:** 10.1002/jev2.12033

**Published:** 2020-11-29

**Authors:** Vivian V.T. Nguyen, Kenneth W. Witwer, Marianne C. Verhaar, Dirk Strunk, Bas W.M. van Balkom

**Affiliations:** ^1^ Department of Nephrology and Hypertension UMC Utrecht Utrecht The Netherlands; ^2^ Department of Molecular and Comparative Pathobiology Department of Neurology The Johns Hopkins University School of Medicine Baltimore Maryland USA; ^3^ Spinal Cord Injury and Tissue Regeneration Center Salzburg (SCI‐TReCS) Cell Therapy Institute Paracelsus Medical University Salzburg Austria

**Keywords:** clinical translation, disease models, organoids, potency, standardization

## Abstract

An important aspect in the development of extracellular vesicle (EV) therapeutics is identifying and quantifying the key features defining their identity, purity, sterility, potency and stability to ensure batch‐to‐batch reproducibility of their therapeutic efficacy. Apart from EV‐inherent features, therapeutic efficacy depends on a variety of additional parameters, like dosing, frequency of application, and administration route, some of which can be addressed only in clinical trials. Before initiating clinical trials, EV‐inherent features should be tested in well‐standardized quantitative assays *in vitro* or in appropriate animal models *in vivo*. Ideally, such assays would predict if a particular EV preparation has the potential to achieve its intended therapeutic effects, and could be further developed into formal potency assays as published by the International Council for Harmonization of Technical Requirements for Pharmaceuticals for Human Use guidelines. Furthermore, such assays should facilitate the comparison of EV preparations produced in different batches, on different manufacturing platforms or deriving from different cell sources. For now, a wide spectrum of *in vitro* and *in vivo* assays has been used to interrogate the therapeutic functions of EVs. However, many cannot accurately predict therapeutic potential. Indeed, several unique challenges make it difficult to set up reliable assays to assess the therapeutic potential of EVs, and to develop such assays into formal potency tests. Here, we discuss challenges and opportunities around *in vitro* and *in vivo* testing of EV therapeutic potential, including the need for harmonization, establishment of formal potency assays and novel developments for functional testing.

## 1 INTRODUCTION

Extracellular vesicles (EVs) have been identified as essential signalling mediators in various physiological and pathophysiological processes through transfer of bioactive molecules such as RNAs and proteins between cells (Lotvall & Valadi, 2007; Stoorvogel, Kleijmeer, Geuze, & Raposo, 2002; Théry, Zitvogel, & Amigorena, 2002; Yáñez‐Mó et al., 2015). EVs can promote therapeutic activities comparable to those ascribed to their donor cells, demonstrating that EVs are important players in the paracrine effects observed in cell therapies (Lai et al., 2010). In animal studies, EVs can promote tissue regeneration by creating a pro‐regenerative immunomodulatory environment by steering endogenous cells to repair affected tissues and by switching immune responses from pro‐inflammatory to tolerogenic. In contrast to cells, EVs are non‐self‐replicating and can, if necessary, be sterile‐filtered (0.22 μm), cryopreserved (Kusuma et al., 2018) or freeze‐dried (Bari et al., 2019), allowing standardized, off‐the‐shelf regenerative therapies. Despite demonstration of the preclinical and clinical positive effects of EVs, broad translation is still limited due to several hurdles (Witwer et al., 2019), including (1) heterogeneity in EV‐preparation procedures, (2) absence of uniform quality control (QC) criteria, and (3) necessity of alignment with national and international regulatory guidelines to reach clinical testing (Lener et al., 2015; Reiner et al., 2017). A recent ‘Call for Action’ underscored that an integrated international collaborative approach is required for successful translation of EVs to clinical application (Roura & Bayes‐Genis, 2019). In light of this call, we address the assessment of the quality, efficacy and therapeutic dose of EVs, and provide arguments for the use of harmonized assays to describe these aspects. We highlight the difference between formal potency assays and functional assays to assess the potential of EVs to have a therapeutic effect, and discuss aspects of various experimental approaches to assess EV functionality.

## 2 EV EFFICACY IN THERAPEUTIC APPLICATIONS

EVs have been applied in many pre‐clinical disease models and in humans (Escudier et al., 2005; Kordelas et al., 2014; Nassar et al., 2016). Therapeutic targets include malignancies like melanoma and head and neck cancer, immunological diseases like arthritis and graft versus host disease, and degenerative kidney, heart and liver diseases (Gatti et al., 2011; Kordelas et al., 2014; Lai et al., 2010; Li et al., 2013). Despite reported evidence for therapeutic effects and increasing adherence to EV preparation and characterization guidelines such as MISEV2018 (Théry et al., 2018), it remains difficult to compare results between studies, EV batches and individual experiments. This is particularly due to the different methods of EV dose calculation, ranging from protein‐ or producer cell equivalents to EV (or particle) numbers. Also, procedures for EV production, including separation protocols, purification, EV donor cell characteristics, cell culture conditions, vary considerably (Borger et al., 2017). If doses cannot be compared, information on EV preparation and application might not be sufficient to estimate the impact of EVs as a therapeutic.

There is an urgent need for a consensus to assess the *potential* of EVs to elicit specific effects, whether or not directly linked to a therapeutic effect for a defined target disease. Functional assays assess the molecular and physiological effects a certain EV preparation has on target cells, organoids, organs or organisms, and may or may not be linked to the potential of an EV preparation to have a specific therapeutic effect. This effect can be assessed by *in vitro* or *in vivo* quantitative assays, allowing comparisons between different EV preparations and doses (Yáñez‐Mó et al., 2015). Functional assays are not necessarily considered *potency* assays as defined by the International Council for Harmonisation of Technical Requirements for Pharmaceuticals for Human Use (ICH) guidelines, European Medicines Agency, and the US Food and Drug Administration (FDA). These regulations define potency as the specific ability or capacity of a product to effect a given result, as indicated by appropriate laboratory tests or by adequately controlled clinical data obtained through the administration of the product in the manner intended (EMA, FDA) (Box 1, ICH).

**Box 1 Definitions and terms**

*EV function* refers to the molecular and physiological effects a certain EV preparation has on target cells, organoids, organs or organisms, and may be linked to the potential of an EV preparation to have a therapeutic effect. This effect can be assessed by *in vitro* or *in vivo* quantitative assays, allowing comparisons between different EV preparations and doses (Yáñez‐Mó et al., 2015).

*Efficacy*, in pharmacy, is the maximum response achievable with a dosed agent, and in medicine, is the capacity for beneficial observation of a given intervention (Holford & Sheiner, 1981). The definition is regulated differently among organizations (Santos et al., 2017).

*Potency* is the specific ability or capacity of a product to effect a given result, as indicated by appropriate laboratory tests or by adequately controlled clinical data obtained through the administration of the product in the manner intended (FDA).

*Potency assays* comprise biological (*in vitro* or *in vivo*) assays, or non‐biological (surrogate) tests, or a combinatorial test matrix, selected for each individual product to indicate its potency, and based on a defined biological effect as close as possible to the molecular mechanism(s) and the clinical response (FDA).

*Biological assays*, or bioassays, are quantitative assays that can measure the specific ability to effect a given result of a product's active ingredient(s) within a living biological system. They include *in vivo* animal studies, and *in vitro* organ, tissue or cell culture systems, or any combination of these (FDA).

*Non‐biological in vitro assays*, or analytical assays, provide surrogate measurements of biological activity by evaluating biochemical and/or molecular characteristics of a product. Only if the surrogate measurements can be substantiated by correlation to a relevant biological activity, these characteristics may be used to demonstrate a product's potency (FDA).

*Limit of detection* (LOD) relates to the sensitivity and accuracy of an assay, and describes the lowest value or concentration which can reliably distinguished from the background. Related, the Limit of Blank (LOB), describes the accuracy by which a true negative control can be measured. The Limit of Quantification (LOQ) describes the range of measurements between the blank and the maximum value at which reliable quantitative values can be obtained from an assay (Armbruster & Pry, 2008).

*The Z factor* (Z’) is a value reflecting the quality of an assay and is defined by the means (μ) and standard deviations (δ) of both the positive (*p*) and the negative (*n*) controls (μp, μn; δp, δn), respectively. It is calculated as: Z’ = 1 ‐ $\frac{3(\delta p+\delta n)}{\left|\mu p-\mu n\right|}$. Good assays should have a Z’ value between 0.5 and 1 (Zhang, Chung, & Oldenburg, 1999).

*Reference material* can be used for standardization of functional assays and EV preparation, allowing comparison of the functionality, or potency, of different EV preparations, either between different labs or between different EV batches (Geeurickx et al., 2019).

*Multi‐organ on a chip* models allow mimicking of physiological interactions between (human) organ representatives, such as organoids, primary cells or cell lines, cultured in separate organ chambers connected through a microfluidic system (Van Den Berg, Mummery, Passier, & Van Der Meer, 2019).

*Organoids* are three‐dimensional organotypic structures derived from (mature) stem/progenitor cells. They retain their physiological and genomic identity over many culturing passages and allow for *in vitro* studies of organ development, tissue homeostasis and disease (Drost & Clevers, 2017).

**FIGURE 1:**
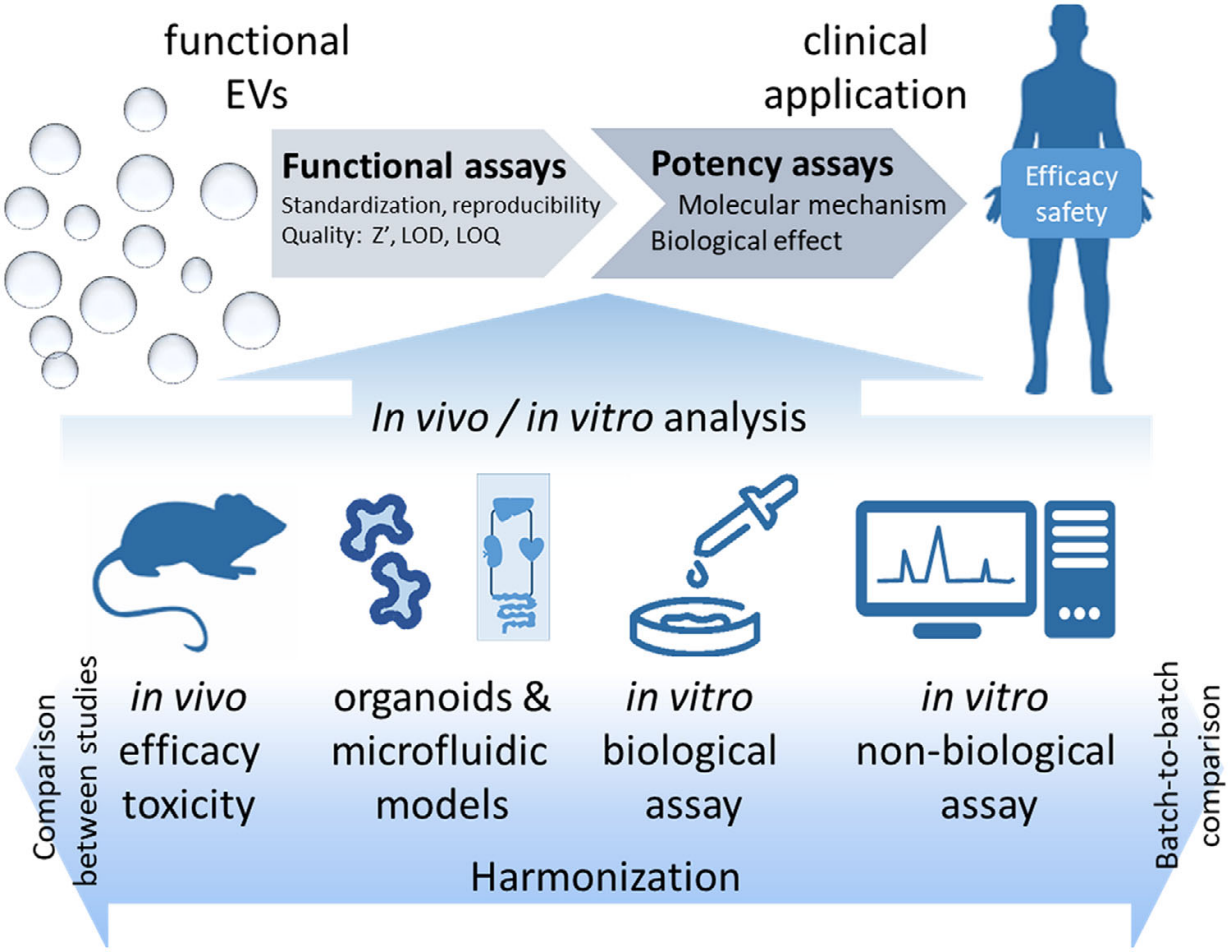
From functional assays to potency assays. The potential of EVs to elicit a specific therapeutic effect can be assessed by *in vivo* or *in vitro* assays. Harmonization of such functional assays will allow comparison of results between experiments and EV preparations. Certain functional assays may also be or be developed into potency assays

According to a formal definition, *potency assays* consist of biological (*in vitro* or *in vivo*) or non‐biological (surrogate) tests, or a combinatorial test matrix, selected for each individual product to indicate its potency, and based on a defined biological effect as close as possible to the known molecular mechanism(s) and clinical response(s) (FDA). Hence, their outcomes are representative of the therapeutic effect and independent of the EV preparation procedure, which not only allows comparisons between studies, but also between different batches of EV preparations. For global harmonization of studies of therapeutic EVs, it is essential to define functional units based on functional assays – not necessarily qualified as potency assays. Functional units describe a quantifiable effect of a certain dose of EVs (expressed as EV number, volume or cell equivalent, i.e.) and are based on assays that can be reproducibly and uniformly implemented. These tests should ideally represent an aspect of the mode of action (MoA) *in vivo*. Thus, such assays should assess the *potential* of an EV preparation to elicit a quantifiable effect related to the target disease, but should not be considered formal potency assays (Figure 1).

## 3 FUNCTIONAL ASSAYS EVALUATING THE THERAPEUTIC POTENTIAL OF EVs

Rigorous functional and safety testing must precede clinical trials for approval of EV‐based therapeutics. Critically, in translating EV‐based therapeutics, quality and safety of EV batches need to be monitored. For both research and regulatory purposes, it is desired to have uniformly accepted, representative assays in place to assess these aspects. As per FDA recommendations, the therapeutic capacity (*potency*) and safety of an EV preparation are assessed through biological or non‐biological potency and safety assays (FDA). There are currently shortcomings in the reporting of *in vitro* assays used to study therapeutic EV‐based products, from quality control to physiological and molecular MoA. Although many assays assessing these aspects have been described, the development of formal potency assays is an unmet need.

By the ‘FDA Guidance for Industry ‐ Potency Tests for Cellular and Gene Therapy Products’, potency is defined as ‘the specific ability or capacity of the product, as indicated by appropriate laboratory tests or by adequately controlled clinical data obtained through the administration of the product in the manner intended, to effect a given result’. This definition also requires that ‘tests for potency shall consist of either *in vitro* or *in vivo* tests, or both, which have been specifically designed for each product so as to indicate its potency in a manner adequate to satisfy the interpretation of potency’ (FDA) (Box 1). Many different potency assays are in place to assess specific characteristics of pharmaceutical products, including assays to determine biological activity, toxicity or physical aspects like solubility (Chung et al., 2004; Mcanally, Vicchiarelli, Siddiquee, & Smith, 2012; Strickley, 2004; White, 2000; Yan & Caldwell, 2001). The use of a combination of potency assays, an assay matrix, may be required to meet all criteria. Potency assays can be biological or non‐biological and should (1) indicate therapeutic strength, (2) be quantitative, (3) include reference materials and (4) verify the identity of the preparation. In addition, for clinical manufacturing, potency assays should (5) provide data for product release, (6) meet predefined acceptance/rejection criteria, (7) provide basis for batch documentation and (8) meet labelling requirements (FDA).

EV‐based therapies in clinical application may be dependent on dose and route of administration. It is critically important to identify the optimal dose, both to prevent potential adverse effects of a surplus dose as well as to be as cost‐effective as possible. Therefore, a potency assay for EVs must evaluate the magnitude of a response to a certain dosage of EVs in *in vivo* or (biological or non‐biological) *in vitro* models. The potency assay does not necessarily represent the underlying mechanism of the targeted pathophysiological or molecular pathways. It is expected that no single test adequately measures all product attributes that predict clinical efficacy. However, combinations of *in vitro* and *in vivo* assays can form a solid basis for appropriate potency assays (FDA).

To assess EV potency, ‘a matrix of functional assays’ might thus be performed as a validation process. Ideally, the potency result should be expressed as a ‘Functional Unit’, which is a dose of EVs at which a specific, quantifiable (biological) response is elicited. An efficient clinical dose is strongly dependent on the targeted disease and the potency of EVs. Therefore, potency assays and combinations thereof will be specific for each therapeutic application.

Historically, chemically synthesized molecules could be measured by ‘Mass Units’. Challenges tend to be much greater because cell‐based products, including EVs, are much more complex and heterogeneous. Thus, Mass Unit is not a sufficient validation of the functional activity for biologicals. For instance, *insulin* has two measurement units: (1) System International (SI) unit (pmol/l) as a mass unit and (2) the conventional international unit (IU/U) that is based on bio‐efficacy. The history of insulin measurement units has changed several times since its discovery in 1922 (Knopp, Holder‐Pearson, & Chase, 2019). In this context, specific potency assays for EVs should be established, capable of predicting a clinical response. Although, EVs have been investigated for their various therapeutic actions, such as cytoprotection, vasculogenesis and angiogenesis, immunomodulation, endogenous regeneration, antifibrotic effects, and metabolic effects (Wiklander, Brennan, Lötvall, Breakefield, & El Andaloussi, 2019; Lener et al., 2015), a typical definition of potency for a pharmaceutical compound may not work for EVs.

### 3.1 Conventional biological *in vitro* assays

Rigorous *in vitro* functional studies can provide evidence for EV potency. Hao et al. investigated the ability of bone marrow stromal cell‐derived EVs to downregulate the expression of multidrug resistance protein 1 (MRP1). They showed that EVs decreased MRP1 activity in a dose‐dependent manner and confirmed their findings in mouse experiments *in vivo* (Hao et al., 2019). Choi et al. demonstrated a dose‐dependent stimulatory effect of EVs in *in vitro* angiogenesis assays. These EVs did not show anti‐apoptotic effects in endothelial cells *in vitro* but improved recovery from acute kidney injury, accompanied by increased renal capillary density (Choi et al., 2014). It is interesting to note that in both examples, the presumed cellular and the molecular MoA(s) were represented by the *in vitro* assays. The effect on acute kidney injury described by Choi et al. seems to involve more than one mechanism, as reflected by different *in vitro* assays. This raises the question of whether different MoA aspects should be addressed with different assays. In this particular study, cell proliferation, apoptosis and stimulation of angiogenesis after EV treatment were assessed. As earlier studies had demonstrated that EV‐mediated prevention of cell cycle arrest allowed an angiogenic program in EV target cells, the effects observed in the different assays might be related to each other (van Balkom et al., 2013). Complementary assays are available determining effects on cell proliferation, regeneration and immune modulation (Ketterl et al., 2015; Pachler et al., 2017).

Biological *in vitro* experiments have provided a comprehensive basis for subsequent *in vivo* experiments. Many cell lines employed in these readouts are well standardized but genetically modified and commonly cultured in a two‐dimensional environment, and therefore morphologically and genetically different from their tissue of origin. A growing number of studies have meanwhile used primary cells or patient organoids for compound and therapeutic EV testing (Dunne, Jowett, & Rees, 2009; Dekkers et al., 2013; Mentkowski, Mursleen, Snitzer, Euscher, & Lang, 2020). In contrast to cell lines, primary cells have a limited lifespan and expansion capacity, and are less easy to maintain, often requiring supplements that are not standard in ‘conventional’ media. Furthermore, primary cells represent individual patient variability. Human organoid cultures were reported to overcome these limitations, and appear to be a promising platform for future drug testing (Drost & Clevers, 2017). Disease modelling using iPSCs and iPSC‐derived organoids will further augment such opportunities (Dutta, Heo, & Clevers, 2017).

### 3.2 Advanced microfluidic‐based *in vitro* models

Recent developments in microfluidics and 2D/3D cell‐ and organoid‐based applications can model the complexity of an organism using organoid combinations in ‘multi‐organ‐on‐a‐chip’ (MOC) systems (Bauer et al., 2017). A great advantage of such systems is that they allow the use of human cells to represent relevant organs (including organoids derived from induced pluripotent stem cells, iPSCs), and their physiological interaction can be mimicked by co‐culturing or through microfluidic connections. Such models thus provide a semi‐systemic platform on a human background and provide valuable alternatives for animal experiments. Using MOCs, information about disease mechanisms, organ‐organ‐interactions and potential EV‐based therapeutics in a human context can be obtained *in vitro*. For example, in a combined system of pancreatic islets and liver spheroids coupled via microfluidic circulation, physiological insulin‐regulated sugar metabolism could be mimicked (Bauer et al., 2017). Besides physiological interactions, MOC systems allow modelling of diseases, such as diabetic nephropathy, to gain insight into disease mechanisms and explore potential therapeutics (Wang et al., 2017). MOC models may also provide information on biodistribution of EVs after systemic application. A combination of kidney and liver representatives in such a system recapitulated the preferential accumulation of cancer cell‐derived EVs in the liver (Tian et al., 2020). Although the added complexity and the use of human organ models allow a stringent pre‐selection of candidate drugs before advancing to animal models (Skardal, Shupe, & Atala, 2016), the true value of MOC models should, similar to traditional *in vitro* models, be confirmed *in vivo*. Animal models often do not fully recapitulate their human counterparts, resulting in false positives (effective in animals, not in humans) and false negatives (not effective in animals, but may be effective in humans) (Mak, Evaniew, & Ghert, 2014). Rather than using animal models for validation of *in vitro* data, the growing amount of clinical and experimental data stored in various curated databases allow *in silico* modelling of human physiology. This enables computational frameworks to integrate experimental data and computational models to translate *in vitro* findings to relevant clinical situations.

Documented parameters, including pharmacodynamics, gene function and expression data, allow quantitative systems pharmacology (QSP) and physiologically‐based pharmacokinetic (PBPK) (Polak, Tylutki, Holbrook, & Wiśniowska, 2019; Gadkar, Kirouac, Parrott, & Ramanujan, 2016) modelling of direct and indirect effects of diverse drugs, including EVs, in a systemic manner. This permits intra‐ and extrapolation of therapy responses and the influence of organs that are not part of the MOC system. Combining data from MOC systems with *in silico* modelling builds an integrated framework in which MOC data inform the *in silico* models, and *in silico* models in turn provide cues for further experimental design, allowing validation (Maass et al., 2019). As such, the use of MOC systems is becoming more popular, and many academic research groups and companies are developing disease‐specific MOC models or combining more organs on a chip, aiming for the ultimate ‘human‐on‐a‐chip’. A major challenge in the development of such systems remain the different growth conditions and metabolic requirements of the different organ representatives. MOC systems could thus provide attractive platforms for functional EV assays. The bear the potential to play a particular role in the reduction and refinement, and in some cases even replacement (3R) of animal experiments (Cavero, Guillon, & Holzgrefe, 2019; Marx et al., 2012).

### 3.3 Biological *in vivo* assays – V function and safety analysis

*In vivo* models can cover multiple aspects of EV applicability, from dosing and administration route to therapeutic effect and potential side effects. Despite advances in MOC models, information obtained from *in vivo* explorations contributes to the deeper understanding and applicability of EV‐based therapies. No other system is able to provide information about the distribution of EVs in the body, circulation time, targeting organs. It has been demonstrated that systemically administered EVs accumulated in spleen and liver within 30 min of IV injection, and were cleared by the hepatic and renal pathways (Faruqu et al., 2019).

Dosing regimens for therapeutic EVs can be determined using different *in vivo* models, considering EV circulation time, clearance rate and therapeutic effect, towards finding the minimal effective and optimal therapeutic doses. Interaction with the immune system provides information about EV dose, administration route and the potential need for repeated administration. Macrophages can remove exogenously administered EVs (Imai et al., 2015). Despite the risk of precipitating immune responses, HEK293T‐derived EVs, even when administered multiple times over a 3‐week period, did not evoke measurable xeno‐immunity in immune‐competent C57BL/6 mice (Zhu et al., 2017). Repeated doses of up to 20 daily injections of rat bone marrow stromal cell‐derived EVs in a rat model of acute kidney disease did not trigger an adverse immune response, contributing to the current view of reduced immunogenicity of EVs (Reis et al., 2012). Repeated applications of stromal or dendritic cell‐derived EVs in humans were also demonstrated to be safe (Escudier et al., 2005; Kordelas et al., 2014).

### 3.4 Advances in *in vivo* models for functional EV analysis

Recent advances in intravital live imaging have enabled the visualization of tumour‐derived EVs in mice (Zomer et al., 2015). Zebrafish have attracted great attention as a potential model to study EVs *in vivo* due to a combination of small size, rapid development, low cost, a mature immune system and optical transparency of the chorion and embryo. Zebrafish are suited for *in vivo* imaging with high‐resolution optical microscopy at the in toto scale as demonstrated by organ‐to‐organ transfer of CD63‐pHluorin reporter‐containing EVs in zebrafish embryos (Verweij et al., 2019). Furthermore, the zebrafish model can also provide a complex vascular network where EV circulation and dissemination can be studied (Hyenne et al., 2019).

The chick chorioallantoic membrane (CAM) represents an embryonic *in vivo* model focusing on angiogenesis in a transparent, highly vascularized extraembryonic membrane. With low cost of experiments, simplicity, and a transparent vasculature, the CAM model is commonly used as an *in vivo* model for (anti‐)angiogenesis studies and has demonstrated its usefulness in EV research (Ribatti, Annese, & Tamma, 2020). EVs derived from cardiomyocytes cultured under hypoxic conditions were found to increase CAM vascular density by 30% compared with EVs from cardiomyocytes cultured under control conditions (Ribeiro‐Rodrigues et al., 2017). In another study, EVs, in contrast to their donor stromal cells, could not stimulate blood vessel formation in this assay (Merckx et al., 2020). These advanced *in vivo* models, combined with high resolution imaging, provide opportunities to shed light on real‐life physiological roles of EVs.

### 3.5 Non‐biological assays

Non‐biological assays are especially valuable to determine whether a particular EV preparation – or batch – meets specific characteristics, like the presence of specific proteins, RNAs, lipids or enzymatic activity, either directly or indirectly (FDA). For example, several RNAs have been described to be crucial components of EVs to elicit a therapeutic effect. miR‐21 in stromal cell‐derived EVs elicits cardio‐protective effect through the PTEN/PI3K/Akt axis (Luther et al., 2018; Shi et al., 2018). Analysis of proteins can be done, e.g., by immunoblotting (Dai et al., 2008) or by flow cytometry (Wiklander et al., 2018). Using biological *in vivo* and *in vitro* assays to assess the functionality of EVs, or any therapeutic, may not always be feasible because of technical or product‐related limitations, like limited batch size or donor‐to‐donor variation in case of autologous products. In such cases, it may be desirable to design *in vitro* non‐biological tests as a means of quality control. Non‐biological assays require knowledge of the MoA and allow assessment of physical or molecular characteristics of the EV preparation (FDA). This is illustrated in a phase I clinical trial on the application of ascites‐derived EVs for colorectal cancer treatment, in which the abundance of major histocompatibility class I (MHC‐I) and heat shock cognate 71 kDa protein (HSC70), both known to be important for the antitumor effect, were assessed using immunoblotting (Dai et al., 2008).

One of the earliest reports on EVs described the analysis of enzymatic activity, showing that cellular ecto‐5′‐nucleotidase activity is released in association with EVs (Trams, Lauter, Salem, & Heine, 1981). The collagen crosslinking activity of lysyl oxidase‐like 2, associated to the outer surface of endothelial cell‐derived EVs, could be determined using both biological and non‐biological assays (de Jong, van Balkom, Gremmels, & Verhaar, 2016). Furthermore, the presence or absence of lipids in the EV membrane can be assessed (Osteikoetxea et al., 2015). Lipids in EV membranes are involved in EV uptake by target cells (Matsumoto et al., 2017) and are target for strategies to enhance circulation time and EV targeting (Kooijmans, Gitz‐Francois, Schiffelers, & Vader, 2018).

Distinguishing a biological assay, performed in living systems *in vitro*, from non‐bioassay methods can be difficult in some contexts. Therefore, non‐biological assays like quantitative flow cytometry, enzyme‐linked immunosorbant assays, molecular profiling (reverse transcription polymerase chain reaction, quantitative polymerase chain reaction, microarray) or biochemical assays (e.g., protein binding, enzymatic reactions), all analyses outside of living systems, are broadly referred as ‘analytical assays’ (FDA).

### 3.6 Impact of EV quantification on functional assays

Quantity of EVs is one possible denominator for function and potency assay results, yet counting EVs remains a major challenge. With the advent of novel technologies, absolute quantification of actual EV amounts and their heterogeneity has become feasible for the first time, and may be of utmost relevance for normalization of inputs. To date, applied EV amounts have often been semi‐quantitative at best, in the form of protein amount, number of particles, quantitative electron microscopy or an estimate of the number of source cells from which the EVs were derived (Giebel & Helmbrecht, 2017; Linares, Tan, Gounou, & Brisson, 2017; Maas, Broekman, & de Vrij, 2017).

Protein assays are reproducible and simple to perform. However, in many EV preparations, most proteins are not located within or associated with the EVs. These extra‐vesicular proteins may be soluble: components of the cell culture media or released by cells along with EVs. Since many EV separation methods only incompletely remove proteins, protein concentration measurements, on their own, convey little information about the actual number of EVs. Another approach is to measure the particle number in preparations using nanoparticle tracking (Sokolova et al., 2011) or resistive pulse sensing (Giebel & Helmbrecht, 2017). These technologies can count and size particles within certain ranges. The results must not be misinterpreted as EV counts, though, because these methods cannot immediately distinguish protein aggregates, salt crystals, or lipoproteins from EVs. Thus, like protein concentration, particle counts alone do not reveal the actual EV content of a preparation. The number of source cells at the time of culture initiation or harvest is a third method for normalizing EV input. However, cell passage numbers, different growth conditions, and harvesting procedure variability impact both the quantity and quality of EV output. Any variability in post‐harvesting steps could also invalidate even the best efforts to standardize growth and harvesting. Considering all possible sources of inaccuracy with these measurements, it is unsurprising to observe tremendous diversity among published studies.

One approach for normalization is to use multiple measures in a ratio or ratios. Protein‐to‐lipid ratio, for example, might be a useful purity measurement. New technologies have also become available that may enable counting of EVs specifically after binding to a sensor or after fluorescently labelling certain EV markers. Strict standardization of all steps from cell culture to EV separation could make protein amount, particle counts, or source cell numbers more useful normalizers. Ideally, such strict and comprehensive standardization could be applied across labs worldwide, and the introduction of physical EV standards may support such attempts (Geeurickx et al., 2019), as fostered effectively by MISEV guidelines (Lötvall et al., 2014; Théry et al., 2018).

### 3.7 Experimental controls and assay quality

By definition, potency measures various attributes of the product, and is determined by suitable potency assays(s) which link(s) directly to biological activity. A common limitation of functional assays preceding validated potency assays is higher variability, which might prevent detection of differences arising from variable manufacturing processes. Comprehensive internal and external controls are needed to address this problem. Potency or functionality of a single EV product tested by in different locations is best assessed in comparison with an appropriate reference material, for example reference particles or EVs (Geeurickx et al., 2019). An elegant way to determine specificity was illustrated by Fang et al., who demonstrated that iPSC‐derived stromal cell EVs, in contrast with control fibroblast‐derived EVs, could decrease the expression of IL‐9 and IL‐13 in innate lymphoid cells (Fang et al., 2020). Fractionation of the cell culture medium confirmed EVs and not soluble factors as mediating also scar reduction after myocardial infarction (Lai et al., 2010).

For all assays including physical or functional characterization of EVs, three factors are essential for obtaining accurate results: the limit of blank (LOB), limit of detection (LOD), and limit of quantitation (LOQ) (Armbruster & Pry, 2008). They relate to the sensitivity and accuracy of an assay. LOD describes the lowest value or concentration that can be reliably distinguished from background, which in turn can be deduced from the variance in the blank measurements, the LOB. In principle, assays are able to provide values between zero and a certain maximum; however, due to the LOB and LOD, it is almost impossible to measure a true zero. The range within which reliable results can be determined starts at the LOQ.

Monitoring the quality of functional as well as potency assays is crucial. There are numerous statistical methods to measure the quality of an assay. The Z factor (Z’) is commonly used, especially for non‐biological assays, and is defined by the means (μ) and standard deviations (δ) of both the positive (*p*) and the negative (*n*) controls (μ_p_, μ_n_; δ_p_, δ_n_), respectively. It is calculated as: Z’ = 1 ‐ $\frac{3(\delta p+\delta n)}{\left|\mu p-\mu n\right|}$. By this formula, a good Z’ value is indicated by a strong difference between μ_p_ and μ_n_, and the lowest value of δ_p_ and δ_n_. Thus, using Z’ factor to validate assay performance will not only give the information about the quality of the assay, but also discriminate good and bad measurement technologies. The best assays and instrumentations are indicated by the Z´ value between 0.5 and 1. If Z´ value is between zero and 0.5, the assay is borderline, but Z´ values below zero indicate that the assay is not useful for screening purposes. For instance, the reliability and reproducibility of a high‐throughput assay of hepatitis C virus helicase inhibitors using fluorescence‐quenching were validated based on Z’ factor value. In this study, the experimental condition that gave the highest Z’ values, ranging from 0.5 to 1, was chosen as the optimal condition (Tani et al., 2009). The Z’ factor therefore became the standard means for validating quality of plate based‐assays (Hughes, Rees, Kalindjian, & Philpott, 2011) and should also be considered for evaluating EV functional assays.

### 3.8 Heterogeneity in EV studies

The term EV itself encompasses a broad range of vesicles, including exosomes secreted upon the fusion of multivesicular bodies with the plasma membrane, mostly nano‐sized microvesicles or ectosomes, which bud off from the plasma membrane, apoptotic bodies and oncosomes (released by cancer cells) (Willms, Cabañas, Mäger, Wood, & Vader, 2018). Harmonization in the nomenclature of EVs is desired and remains a subject for discussion (Witwer & Théry, 2019). EVs are prepared by different separation methods depending on the EV's physical and molecular characteristics. A gold standard method has not been agreed on, since every method has its own pitfalls and advantages related to feasibility and practicality and to co‐separation of non‐EV particles, proteins, and other entities that may or may not be considered ‘impurities’. For example, RNA, thought to be an important contributor to EV‐mediated intercellular communication (Mateescu et al., 2017; Valadi et al., 2007) was present in only minute amounts in EVs captured with cholera toxin‐loaded beads, despite high recovery of small EV (sEV) proteins. In contrast, capture with Shiga toxin‐loaded beads yielded high RNA content but with limited recovery of typical sEV proteins (Lai et al., 2016). As another example for variability of results, laborious density gradient centrifugation/ultrafiltration procedure together with size‐exclusion chromatography resulted in highly variable EV purification from primary body fluids or serum‐containing media partly due to variable lipoprotein co‐enrichment (Corso et al., 2017; Karimi et al., 2018; Vergauwen et al., 2017). Despite efficient elimination of non‐EV proteins by these methods, purity was still variable since high and low density lipo‐proteins (HDL, LDL) in serum can self‐aggregate forming larger particles, thus sharing similar physical characteristic with EVs, a well‐recognized phenomenon that requires specific analysis and separation techniques if high purity of EVs is pursued (Simonsen, 2017; Sódar et al., 2016). Ideally, EV functional assays would assist in selecting the most appropriate isolation methods for EVs for a given application. The MISEV guidelines also provide a framework for EV researchers to stimulate harmonization rigor, reproducibility and at the same time provide room for innovation (Lötvall et al., 2014; Théry et al., 2018; Witwer et al., 2017). These guidelines are based on experiences and opinions of the EV research community, and very much depend on transparency of reporting research methodology and obtained data. Online platforms as EV‐TRACK and Vesiclepedia facilitate sharing of methods and aid scientific transparency in the EV field (Kim et al., 2015; Van Deun et al., 2017).

Potency may be assessed using biological *in vivo* and *in vitro* assays, non‐biological assays or a combinatorial assay matrix. *In vivo* disease models obviously provide comprehensive insight. However, *in vivo* models also introduce more variables, as animals, housing conditions and surgical skills differ between and even within groups. Despite great variation between studies in EV cell source, separation method, disease model, dose, etc., comparison is possible to a certain extent. For example, different therapeutic EV preparations were used in the same mouse model for acute kidney injury (AKI). Mesenchymal stromal cell (MSC)‐derived EV (MSC‐EV) dosing was reported as 15 μg (Bruno et al., 2009) or 2.2×10^8^ particles (Collino et al., 2015) – both corresponding to the amount of EVs secreted by 75,000 MSCs overnight. Whereas dosing of liver stem cell EVs was reported as ‘EVs from 3.5×10^5^ or 10×10^5^ cells’, with no difference in therapeutic effect (Sanchez et al., 2014). Although in each case a beneficial effect of EVs was observed, therapeutic efficacy per EV dose differed between studies, possibly reflecting EV batch or experiment(er) variance. Two papers described similar effects of different EV doses (30 μg vs 100 μg) of umbilical cord stromal cell EVs on improvement of kidney function and morphology in a rat AKI model (Ju et al., 2015; Zou et al., 2014), whereas stronger effects were reported in a slightly different AKI model using a 100 μg dose (Gu et al., 2016).

**FIGURE 2:**
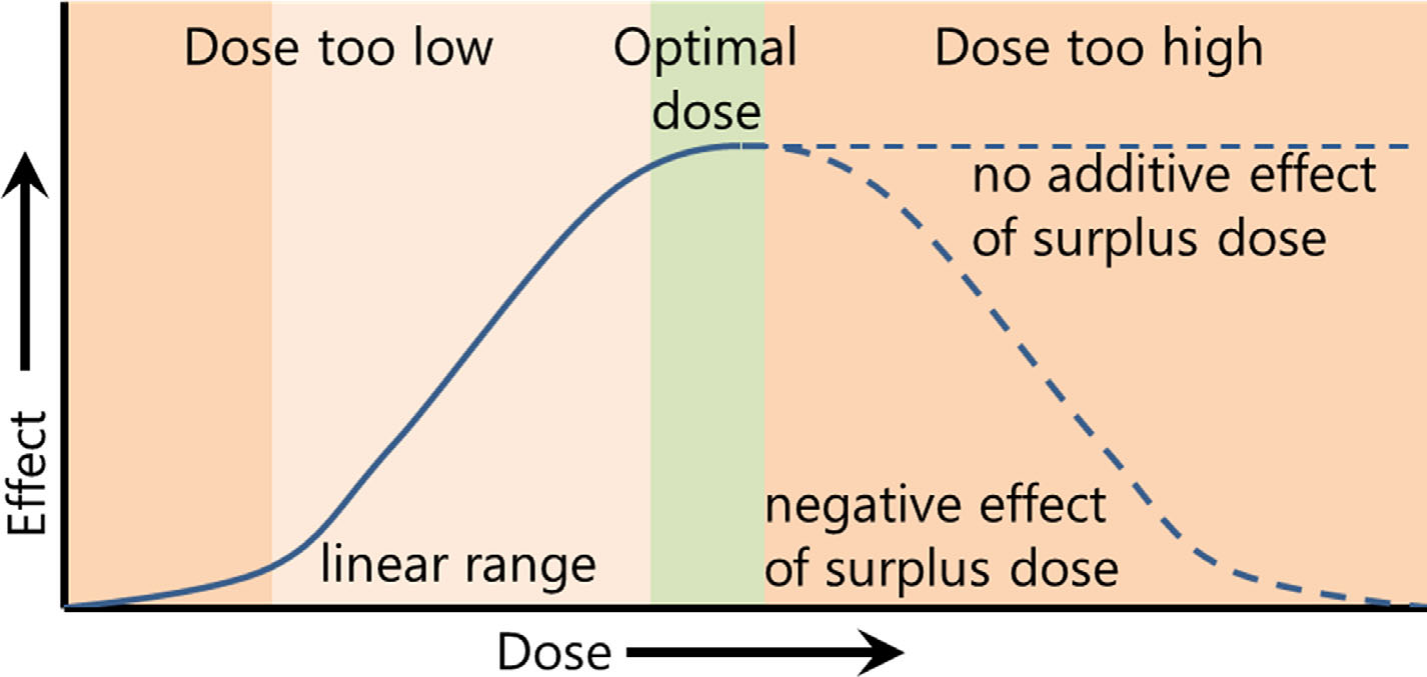
Dose response curve. Precise dosing of therapeutic EVs is essential to achieve optimal therapeutic effects. Only above a certain threshold dose will effects become evident. Increasing doses leads to better effects within a linear range. Determining the optimal dose helps to prevent over‐administration and may also minimize adverse effects

In some studies, *in vitro* data supported progress to an *in vivo* approach, explaining the observations made *in vivo* in a mechanistic manner. Zhang et al. demonstrated that human embryonic stem cell‐derived MCS‐EVs promoted cartilage repair *in vivo* using a rat osteochondral defect model indicating that stimulation of migration and proliferation were part of the MoA (Zhang et al., 2018). Although the *in vitro* studies used different doses of MSC‐EVs, ranging from 1 to 10 μg/ml, only one dose (100 μg per rat) was used in the *in vivo* experiments. The *in vitro* studies were performed with tens of thousands of cells in volumes of 200–300 μl. How do these doses translate to 250‐g rats? The lowest dose in the *in vitro* studies was 1 μg/ml in 300 μl given to 2 × 10^4^ cells, thus 1.5 × 10^–5^ μg/cell. A 250‐gram rat consists of approximately 8×10^15^ cells, making the *in vivo* dose 1.25 × 10^–16^ μg/cell. While this dose appeared rather low, it also assumed equal distribution of EVs to all cells, and this is almost certainly not the case given common distribution patterns of EVs. The liver is the major accumulation site for EVs, but in diseased animals, EVs may also be efficiently retained in affected tissues/organs (Grange et al., 2014; Wen et al., 2019) to enhance local regenerative effects. Systemic immunological and metabolic effects are also likely to contribute to therapeutic effects (Arslan et al., 2013; Harrell, Jovicic, Djonov, Arsenijevic, & Volarevic, 2019; Kordelas et al., 2014). Achieving a complete understanding of all biological and therapeutic is important but challenged by the influence of tissue distribution, complex EV content, and variations between and within EV preparations. These examples illustrate that a given EV dose, as assessed by EV number or μg protein, is not necessarily predictive of a given effect. Dose‐dependent effects differ between EV preparations, disease model and other factors. The fact that similar effects could be achieved using an approximately three‐fold different dose illustrates that either the efficacy of the EVs to induce a certain effect differs between studies, or that the highest or even both doses were above the optimal dose, illustrating the need for improved functional assays (Figure 2).

Several questions thus arise. What is the optimal dose of EVs in a therapeutic setting? How can this dose be determined? Can doses be compared between EV batches prepared at different times or in different laboratories? EVs can be produced from conventional cell lines, which are highly proliferative and easy to maintain, suggesting the development of a master cell bank for EV production. If the potency of produced EVs could be confirmed by reliable assays, the only remaining concern would then be ‘batch‐to‐batch’ deviation. Accurate and reliable assays for quantification and assessment of function are therefore needed to achieve a better understanding of the therapeutic potential of EV preparations.

As one example, an *in vitro* assay for measuring the anti‐inflammatory activity of EVs on a mouse macrophage cell line was recently reported. In this assay, seven preparations of MSC‐derived EVs, released into seven different conditioned media were collected and purified. EV concentrations ranged from 13.1 to 51.2 × 10^9^ particles/ml, and 0.5 × 10^9^ particles/ml were used for assessing the ability of EVs to reduce the macrophage response to lipopolysaccharide. Five of the seven EV samples provided almost the same effect of anti‐inflammatory activity. Only one sample did not affect at the tested concentration, whereas another sample showed dominant activity and completely inhibited the LPS‐stimulated expression of IL‐6. These differences in the anti‐inflammatory activity emphasize the variance of the preparations considered to result from differences in the purification process and storage conditions (Pacienza et al., 2019). In another example, the therapeutic effect of cardiac progenitor cell‐derived EVs was assessed with an *in vitro* apoptosis/viability assay developed in house. Staurosporine was used to induce apoptosis. EVs were added at different concentrations (0.5 and 5 ng/ml for viability test; 3 and 15 ng/ml for angiogenic test). Vehicle and/or EVs from normal human dermal fibroblasts were used as controls. Cells were stained with calcein and propidium iodide and fluorescence was measured as representative for viability and apoptosis, respectively. The pro‐angiogenic activity of EVs at different concentrations was measured assessing tubule formation, visualized by optical microscopy. A quantification of CD31 expression was also performed, showing angiogenesis‐stimulating capacity of the EV preparations (Andriolo et al., 2018).

As a next step in the process, dose–response curves of EVs should be generated representing the diverse function of EVs. Generally, a therapeutic showing an ‘all or nothing response’ may give an inaccurate read‐out. Determining a dose–response curve is essential to estimate a half‐maximum inhibitory concentration to be used to assess and compare potencies among different candidate preparations. A wide range of existing *in vitro* potency assays for pharmaceutical compounds might be applied to EVs; however, EV therapeutics might require further investigation in tailored assays. Selected examples of functional assays for therapeutic EVs are shown in Table 1.

**TABLE 1:** Examples of *in vitro* and *in vivo* EV functional assays

EV effects	Potential measurement	Assays	Platform	References
Cytoprotective	Against harmful mediators/reduce apoptosis and necrosis/improve tissue function	Live/Death	*In vitro/vivo*	Santoso et al. (2020), Liang et al. (2020), Wang, Bonacquisti, Brown, and Nguyen (2020)
		Lactate dehydrogenase assay	*In vitro*	Liang et al. (2020)
		MTT assay	*In vitro*	Wang et al. (2020)
		MTS assay	*In vitro*	Liang et al. (2020)
		CCK‐8	*In vitro*	Lv, Duan, Wang, Gan, and Xu (2020), Xie, Liu, Chen, and Liu (2019)
		Flow Cytometry	*In vitro*	Xie et al. (2019)
		TUNEL assay	*In vitro/vivo*	Santoso et al. (2020), Xie et al. (2019)
Vasculogenesis and Angiogenesis	Enhance tube formation/vessel length/branching length/number of junctions/nodes/meshes	Matrigel plug assay	*In vitro*	van Balkom et al. (2013)
		Hind limb ischemia assay	*In vitro*	van Balkom et al. (2013)
		Matrigel angiogenesis assay	*In vitro*	van Balkom et al. (2013), Zhong et al. (2020)
		Angiogenesis assay	*In vitro/vivo*	Santoso et al. (2020), Wang et al. (2020), Zhong et al. (2020)
		Echocardiography	*In vivo*	Liu et al. (2020)
		Bead sprouting assay	*In vitro*	van Balkom et al. (2013)
Immunomodulation	Inhibit T/B/DC‐cell proliferation. Suppress (pro‐)inflammatory cytokines (TNF‐α/IL‐6/IL‐1β/IFN‐γ/)	T‐cell proliferation assay	*In vitro*	Pachler et al. (2017), Conforti et al. (2014), Wolfers et al. (2001)
		B‐cell proliferation and differentiation assay	*In vitro*	Pachler et al. (2017), Conforti et al. (2014), Wolfers et al. (2001)
		Macrophage polarization	*In vitro*	Wang et al. (2020), Di Trapani et al. (2016)
		Enzyme‐linked absorption assay	*In vitro*	Liang et al. (2020), Conforti et al. (2014)
		(Pro‐)inflammatory cytokines qPCR	*In vitro*	Liang et al. (2020)
		Lymphocyte proliferation assay	*In vitro/vivo*	Shahir et al. (2020)
		Antigen‐presentation assay	*In vitro/vivo*	Wolfers et al. (2001), Vincent‐Schneider (2002)
		Immunological assay	*In vitro/vivo*	Di Trapani et al. (2016), Liu et al. (2020)
Endogenous regeneration	Stimulate resident cells (proliferation/function/macrophage polarization/migration/adhesion/etc.)	Scratch wound migration assay	*In vitro*	van Balkom et al. (2013), Zhong et al. (2020), Heo, Yang, Rhee, and Kang (2020), Vrijsen, Sluijter, and Schuchardt (2010)
		Proliferation Assay	*In vitro*	Zhong et al. (2020), Aucher, Rudnicka, and Davis (2013)
		Trans‐well migration assay	*In vitro*	Zhong et al. (2020), Liu et al. (2020)
Antifibrotic	Inhibit collagen accumulation/α‐SMA expression. Decrease fibrosis/promote tissue function	Histopathology for collagen area	*In vivo*	Rong et al. (2019), Eirin et al. (2017)
		Ishak fibrosis score	*In vivo*	Rong et al. (2019)
		Hydroxyproline	*In vivo*	Rong et al. (2019)
		Malondialdehyte	*In vivo*	Rong et al. (2019)
		Collagen expression Assay	*In vitro*	Wang et al. (2020)
		Immunohistochemistry	*In vivo*	Rong et al. (2019), Eirin et al. (2017)
		Immunological assay	*In vivo*	Di Trapani et al. (2016), Rong et al. (2019)
Uptake and distribution	Engraftment rate/metabolic remodelling (cell proliferation/differentiation/migration/adhesion/autophagy)/promote or inhibit drug uptake/etc.	ELISA	*In vitro/vivo*	Liu et al. (2020)
		CRISPR/Cas9 reporter assay	*In vitro*	de Jong et al. (2020)
		EV uptake assay	*In vitro/vivo*	van Balkom et al. (2013), Zhong et al. (2020), Di Trapani et al. (2016)
		Luminescence‐based bio‐imaging	*In vivo*	Grange et al. (2014)
		Transmission electron microscopy	*In vivo*	Santoso et al. (2020)

## 4 EVs IN CLINICAL STUDIES

Currently, 174 clinical studies testing EVs registered in the U.S. National Library of Medicine's clinicaltrials.gov. However, only 17 studies intend to apply EVs as a therapeutic intervention, of which four have been published (Table 2) (Dai et al., 2008; Escudier et al., 2005; Nassar et al., 2016; Sengupta et al., 2020).

**TABLE 2:** Registered and published interventional clinical EV studies

No.	Disease	Phase	Intervention	Dose	Status	Result	Reference
1	Dystrophic epidermolysis bullosa wounds	I;IIA	AGLE‐102 (MSC‐derived allogenic EVs)	Not provided	Not yet recruiting	Not yet published	NCT04173650
2	Bronchopulmonary dysplasia	I	UNEX‐42 (BM‐MSC‐EVs)	20; 60; 200 pmol phospholid/kg body weight	Recruiting	Not yet published	NCT03857841
3	Cerebrovascular disorders	I;II	miR‐124 transfected MSC‐EVs	200 μg protein	Finished	Not yet published	NCT03384433
4	Ulcer	I	Plasma‐derived EVs	Not provided	Not yet recruiting	Not yet published	NCT02565264
5	Diabetes mellitus type 1	II; III	MSC EVs	1st small EVs: 1.22–1.51 × 10^6^/kg, IV. 2nd microvesicles: 1.22–1.51 × 10^6^/kg, IV	Not yet recruiting	Not yet published	NCT02138331
6	Polycystic ovary syndrome	NA	Ginger and Aloe EVs	Not provided	Recruiting	Not yet published	NCT03493984
7	Pancreatic cancer	I	KRAS G12D siRNA containing MSC‐EVs	Not provided	Not yet recruiting	Not yet published	NCT03608631
8	Macular holes	I	MSC EVs	50 μg; 20 μg MSC EVs in 10 μl PBS	Recruiting	Not yet published (pilot study: Zhang et al., 2018)	NCT03437759
9	CKD	II/ III	MSC EVs	Two doses of 100 μg/kg/dose	Finished	Published	Nassar et al. (2016)
10	Colon cancer	I	Ascites‐derived EVs	100, 200, 300, 500 μg	Finished	Published	Dai et al. (2008)
11	Melanoma	I	Dendritic cell derived EVs	0.13 or 0.40 × 10^14^ EVs	Finished	Published	Escudier et al. (2005)
12	Periodontitis	I	Adipose stem cells derived EVs	Not provided	Recruiting	Not yet published	NCT04270006
13	Coronavirus pneumonia	I	Adipose stem cells derived EVs	2.0×10^8^ EVs/3 ml	Not yet recruiting	Not yet published	NCT04276987
14	Craniofacial neuralgia	NA	Neonatal stem cell products infused EVs	45 mg or 15 mg infused EVs	Not yet recruiting	Not yet published	NCT04202783
15	Refractory depression, anxiety, neurodegenerative dementias	NA	C‐section amniotic fluid derived EVs	15 cc of EVs in 200 cc of normal saline	Not yet recruiting	Not yet published	NCT04202770
16	Multiple organ dysfunction syndrome after surgical repaired for acute type A aortic dissection	NA	MSC EVs	150 mg MSC EVs	Not yet recruiting	Not yet published	NCT04356300
17	COVID‐19	I	MSC EVs	15 ml ExoFlo	Finished	Published	Sengupta et al. (2020)

In the report of the first published clinical trial using EVs, a phase I clinical trial using autologous exosomes pulsed with MAGE 3 peptides for the immunization of stage III/IV melanoma patients, two dose levels of either MHC class II molecules (0.13 vs. 0.40 × 10^14^ molecules) or peptides (10 vs. 100 μg/ml) were tested. The bioactivity of exosomal MHC class II molecules was tested in a biological *in vitro* assay by using a staphylococcal superantigen E (SEE)‐based multiplex bioassay. EVs were first incubated with femtomolar doses of SEE and washed by density cushion. SEE‐harbouring EVs were next pulsed onto Raji cells which then were subjected to Jurkat reporter cell lines that produced IL‐2 in response to SEE. IL‐2 concentrations in the supernatants of the Raji/Jurkat T cells were assessed using a commercial IL‐2 ELISA, showing increased levels after EV treatment. No major (>grade II) toxicity was observed in EV treated patients, demonstrating the safety of EV application (Escudier et al., 2005).

Another phase I clinical trial, in which autologous ascites‐derived exosomes combined with granulocyte/macrophage colony‐stimulating factor (GM‐CSF) were tested as immune therapy for colorectal cancer assessed the potential of the EVs to have a therapeutic effect using a non‐biological *in vitro* assay (Dai et al., 2008). The abundance of major histocompatibility class I MHC‐I and HSC70 molecules, known to be critical for the anti‐tumour immune response, was assessed via immunoblotting for each batch of the isolated autologous ascites‐derived EVs. This trial demonstrated the safety and feasibility of an EV‐based therapy.

A recently published application of MSC‐derived EVs in COVID‐19 patients (Sengupta et al., 2020) could be seen as suggestion the possibility of safe application of EVs. However, it should be noted that clinical trials should be designed and performed with utmost care, even in the case of a global pandemic that requires urgent resolution. These points were raised by representatives of the International Society for Extracellular Vesicles and the International Society for Cell and Gene Therapy (Lim, Giebel, Weiss, Witwer, & Rohde, 2020). In the study in question, limited reporting of EV characterization, dose, *in vitro* and *in vivo* functional assays, and controls results in limited ability to draw conclusions about whether and to what extent EVs may have contributed to the reported outcomes.

Unfortunately, a lack of information on EV preparations is by no means limited to the COVID‐19 trial, suggesting an important opportunity for improvement in the field. In one registered trial (NCT03437759), first posted in 2018, investigators recruited patients with macular holes and significant vision loss. Patients were stratified to receive either umbilical cord blood MSC‐derived EVs, termed ‘exosomes’ (20 or 50 μg in 20 μl PBS), separated by ultracentrifugation, or MSCs (5 × 10^3^ MSCs in 20 μl PBS) in a control group via intravitreal injection. In a pilot study from the same group, five patients treated with MSC‐derived EVs responded well and showed reduction of macular holes as observed in response to MSC treatment. However, it is not clear exactly what the preparation was. While ultracentrifugation‐based EV concentration procedures are common, co‐isolates are predominant, and EV characterization was mainly based on electron microscopy analysis that did not satisfy MISEV criteria. Also, *in vitro* and *in vivo* functional assays to assess the potential therapeutic efficacy of these EVs were lacking in this publication of pilot data (Zhang et al., 2018). In a second study (NCT02138331), the impact of umbilical cord blood‐derived EVs on β cell mass in patients with type I diabetes continues to enrol patients by invitation. The details for this trial have not been updated since May 2014, while the same research group has performed a study using the same preparation to treat chronic kidney disease. The recently published clinical trial using umbilical cord MSC‐derived EVs does not investigate the ability of the isolated EVs to elicit a therapeutic effect, although some basic characterizations were performed to determine the presence of EVs in the preparations (Nassar et al., 2016). While significant clinical improvement was observed in EV‐treated patients, the lack of reported details and poor adherence to MISEV guidelines (Witwer & Théry, 2019) makes it difficult to draw solid conclusions from this study.

In a planned trial (NCT03384433) of intravenous MSC‐derived EVs to treat patients with acute ischemic stroke, allogeneic MSCs are transfected with miR‐124, with the goal of producing miR‐124‐loaded EVs to enhance neurite remodelling and neurogenesis. Details of EV preparation and characterization are lacking, including in previous publications from the research group. Furthermore, the study is now registered as ‘completed’ with a total of only five recruited patients, raising doubts about whether a ‘randomized, placebo‐controlled phase I/II trial’ has been achieved. Another study (NCT03608631) outlines a clinical protocol for treatment of patients with metastatic pancreatic cancer harbouring a KrasG12D mutation with MSC‐derived EVs that contain small interfering RNA against KrasG12D. As for many registered clinical trials, details on EV separation and characterization (functional or otherwise) are unavailable. There is clearly a need to better establish purity and function of EVs if outcomes of future trials are to be attributed to EVs versus co‐isolated entities.

## 5 FUTURE DIRECTIONS

The many options for EV donor‐cell choice and culture methods, EV preparation, characterization, as well as *in vitro* and *in vivo* disease models have enabled great advances in therapeutic EV development. They have also introduced challenges in comparing data between different studies. Besides harmonization of EV nomenclature (Witwer & Théry, 2019) and characterization (Théry et al., 2018), the need for standardization of functional assays has become an important issue (Geeurickx et al., 2019; Roura & Bayes‐Genis, 2019; Witwer et al., 2019). For therapeutic EVs, potency assays are needed to fill this gap. Many *in vivo* and *in vitro* biological and non‐biological assays are currently in use to assess the potency of EVs to elicit a specific therapeutic effect. Although such assays are crucial for understanding the MoA, they are not formally applicable as potency assays. The general use of such assays by laboratories around the world, with or without reference standards (Geeurickx et al., 2019), would allow comparison of experimental outcomes and therapeutic efficacy of EV preparations globally. Like the MISEV2018 guidelines (Théry et al., 2018), the use of standard assays may not be restrictive. Specific therapeutic strategies and personalized medicine approaches encourage researchers to develop advanced models to assess their therapeutic EV preparation. MOC‐ or organoid‐based assays including iPSC technology provide novel tools for EV functional characterization. The potential of such assays to replace *in vivo* experiments may allow for broader, standardized analysis of EV function, independent of variables occurring in animal experiments such as operator skills, animal supplier, age or gender. The advent of novel technologies and an increasingly better understanding of EV functions in health and disease will further contribute to selection of the most appropriate assay formats for designated applications.

## CONFLICT OF INTEREST

The authors do not declare any conflict of interest.

## Data Availability

Not applicable.
